# Universal dispersion of surface plasmons in flat nanostructures

**DOI:** 10.1038/ncomms4604

**Published:** 2014-04-10

**Authors:** Franz-Philipp Schmidt, Harald Ditlbacher, Ulrich Hohenester, Andreas Hohenau, Ferdinand Hofer, Joachim R. Krenn

**Affiliations:** 1 Institute of Physics, University of Graz, Universitätsplatz 5, Graz 8010, Austria; 2 Institute for Electron Microscopy and Nanoanalysis (FELMI), Graz University of Technology, Steyrergasse 17, Graz 8010, Austria

## Abstract

Dimensionality has a significant impact on the optical properties of solid-state nanostructures. For example, dimensionality-dependent carrier confinement in semiconductors leads to the formation of quantum wells, quantum wires and quantum dots. While semiconductor properties are governed by excitonic effects, the optical response of metal nanostructures is dominated by surface plasmons. Here we find that, in contrast to excitonic systems, the mode dispersions in plasmonic structures of different dimensionality are related by simple scaling rules. Employing electron energy loss spectroscopy, we show that the modes of silver nanodisks can be scaled to the surface and edge modes of extended silver thin films. We thereby introduce a general and intuitive ordering scheme for plasmonic excitations with edge and surface modes as the elementary building blocks.

## Abstract (web summary)

The dimensionality of nanostructures is important in determining their properties. Using electron spectroscopy, Schmidt *et al*. systematically study the plasmonic modes in silver nanodisks as they vary their dimensionality, and find that they can be scaled to the film and edge modes of extended silver films.

## Graphical abstract

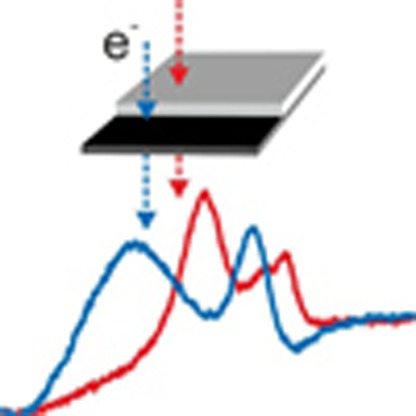

Light can be confined to subdiffraction volumes by binding light fields to nanostructures. Besides being of fundamental interest, this topic holds promise for a variety of photonics applications, such as optical communication and storage^1^ or quantum optics^2^. Surface plasmons (SPs) at metal surfaces, that is, resonant collective electron oscillations coupled to an evanescent light field, play a central role in this quest^3^,^4^. SPs display highly confined modes with accordingly high mode densities that thus interact particularly strong with light. In addition, the spatial and spectral properties of SPs can be easily tuned by controlling the structure geometry. On one hand, this enables to tailor the SP field pattern. On the other hand, the SP resonances can be set within a wide range of visible and infrared frequencies^5^. Improved experimental and theoretical characterization methods have recently considerably widened our insight in the fundamental SP properties. By using scanning optical near-field microscopy, both the intensity and phase patterns of nanoparticle SP modes were imaged in the visible^6^ and infrared spectral ranges^7^. Unprecedented spatial resolution is provided by scanning transmission electron microscopes (STEMs) that, in combination with electron energy loss spectroscopy (EELS)^8^, can probe the (partial) local density of states of plasmonic modes^9^,^10^. This approach was used to map SP modes in nanoparticles^11^,^12^, nanowires^13^ and coupled nanoparticles^14^, enabling even full three-dimensional (3D) reconstruction of field patterns^15^,^16^. In particular, STEM–EELS can probe optically dark modes^17^,^18^, that is, modes with a vanishing net dipole moment, thereby complementing optical methods to map the full modal spectrum of a plasmonic system.

Here we use STEM–EELS on flat metallic nanostructures, as typically produced with electron-beam lithography, to show that plasmonic excitations can be reduced to surface and edge modes as the two elementary building blocks. We probe the specific SP modes sustained by the flat surface and the edge of a silver thin film, comparing an extended system with a straight edge and a nanoparticle (nanodisk) with a radius as small as 50 nm. We find that the nanosdisk modes correspond perfectly to those of the extended film, within measurement error, on applying simple scaling rules, and support our finding with a Drude-like model. In analogy to the hybridization model of plasmon modes^19^, this allows for a simple and intuitive ordering scheme of SPs. In what follows, we systematically work through the SP modes of varying dimensionality (quasi-2D film-like modes, quasi-1D edge-like modes and quasi-0D particle modes), and finally submit our results to the unifying scaling laws.

## Results

### Film plasmons

**Figure 1:**
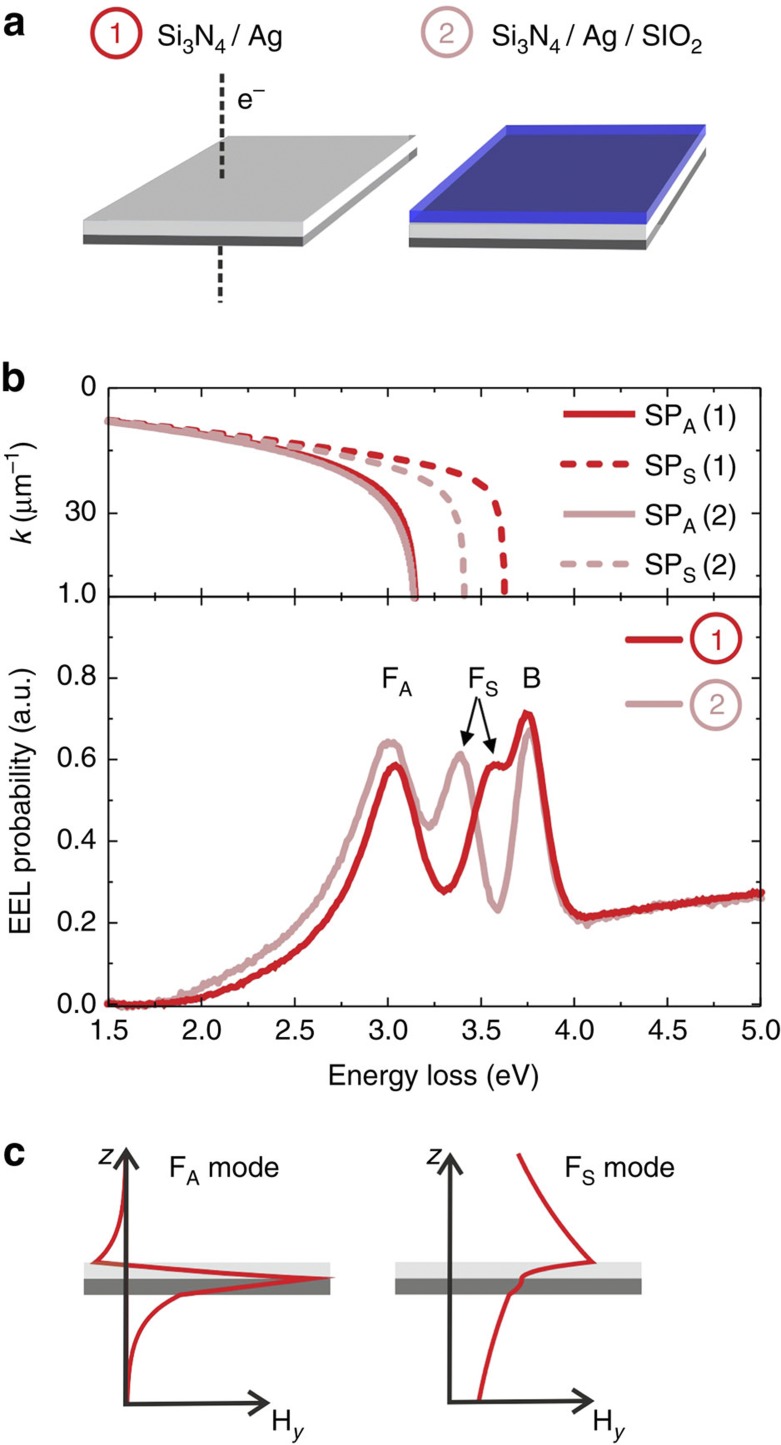
Thin-film SP modes measured by EELS. (**a**) Sketch of the sample geometry, (1) a 50-nm-thick silver film on a 15-nm-thick Si_3_N_4_ substrate, (2) the same with an additional 22-nm-thick SiO_2_ layer. (**b**, top) Calculated dispersion relations of antisymmetric (solid lines) and symmetric (dashed lines) SP modes SP_A_ and SP_S_, respectively, for both sample geometries (1), red and (2), pink. (**b**, bottom) EEL spectra acquired on both systems (1) and (2). (**c**) Calculated magnetic field profile for F_A_ and F_S_ for sample geometry (1).

We start with a 50-nm-thick extended silver film on a 15-nm Si_3_N_4_ membrane (Fig. 1a, left). The EEL spectrum measured with the focused electron beam of the STEM shows three peaks in the energy range from 1.5 to 5 eV (Fig. 1b, red spectrum). Besides the well-known bulk plasmon peak (B) at 3.75 eV^20^, we find two peaks at lower energies that, as we show in the following, are due to film SP modes. Assuming the general case of a coupling of the SP modes at both film interfaces, we label the lower and higher energy modes F_A_ and F_S_, respectively, with the subscripts standing for the antisymmetric (A) and symmetric (S) nature of the transversal magnetic field component. As evident from the calculated field profiles in Fig. 1c, the field strength maxima of F_S_ and F_A_ are in the silver/vacuum and Si_3_N_4_/silver interfaces, respectively, with only weak mutual coupling for the chosen film thickness. We corroborate this interpretation with the EEL spectrum acquired from a 50-nm-thick silver film covered with a 22-nm-thick SiO_2_ layer (Fig. 1a, right). As illustrated by the pink spectrum in Fig. 1b, the SP mode F_A_ (as well as the bulk mode B) is not affected by the cover layer. In contrast, the F_S_ mode at the upper silver interface exhibits a distinct energy shift from 3.6 to 3.3 eV due to the presence of the SiO_2_ layer.

The spectral position of the film mode peaks can be assigned to the asymptotic branches of the corresponding dispersion relations. This is illustrated in the upper panel of Fig. 1b showing calculated dispersion relations in a *k*(*E*) plot (*k* wavenumber, *E* energy) for the layer systems with and without SiO_2_ cover layer for both symmetric and antisymmetric coupling. The asymptotic SP energies at large *k*-values correspond well to the EEL peak energies (Fig. 1b, lower panel) that reflect the SP density of states. We observe, however, a small systematic shift of the peak maximum towards lower energy values, which we attribute to the strong SP damping at large *k*-values^21^ and the accordingly reduced interaction strength with the electron beam.

### Edge plasmons

**Figure 2:**
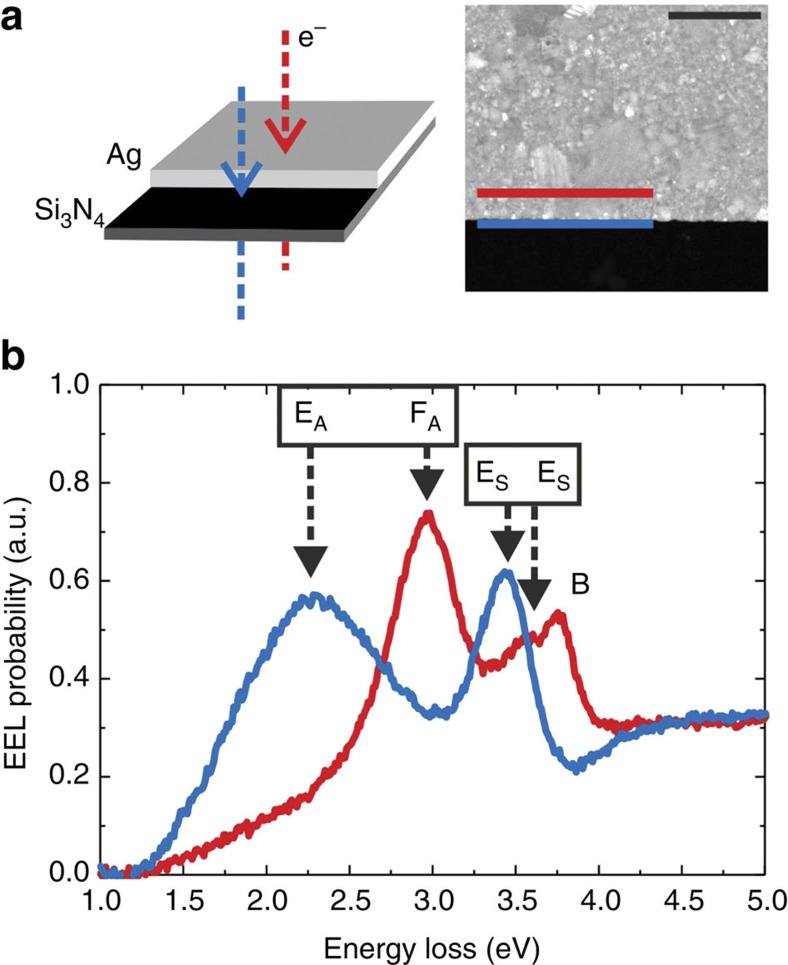
Film versus edge plasmons. (**a**) Sketch (left) and TEM image (right, scale bar in upper corner is 500 nm) of the edge of a 30-nm-thick silver film on a 15-nm-thick Si_3_N_4_ substrate. The dashed arrows in the sketch indicate the electron trajectories transversing (red) and bypassing (blue) the film, the red and blue solid lines in the image mark the sites where the spectra in **b** were measured. (**b**) EEL spectra acquired on the film 45 nm away from the edge (red) and 15 nm outside the edge (blue), as marked in **a**.

Next, we analyse a 30-nm-thick silver film with lithographically defined straight edges and an overall dimension of 100 × 100 μm^2^ on a 15-nm-thick Si_3_N_4_ membrane. Great care was taken to produce defect-free edges for which the reduced film thickness turned out advantageous, while leading to no significant increase in coupling between both film interfaces as compared with the case in Fig. 1. We note that a thickness of 30 nm is maintained for all considered structures in the following. While a thickness dependence is not included in the scaling laws that we discuss, we show in Supplementary Fig. 1 that such a dependence is only weak for thickness range relevant for flat lithographed structures (≥30 nm). A sketch and a TEM image of the sample are shown in Fig. 2a. The EEL spectrum acquired on the film (integrated along the red line in Fig. 2a, distance to edge 45 nm) as plotted in Fig. 2b fits well with the corresponding spectrum in Fig. 1b. The higher relative intensity of the F_A_ mode that is mainly localized at the substrate/silver interface is due to the reduced silver thickness and further illustrates our correct mode assignment. In the EEL spectrum taken outside the silver film at a distance of 15 nm from the edge (integrated along the blue line in Fig. 2a), neither the bulk plasmon peak nor the two F peaks are present. However, two new peaks emerge at loss energies of 2.3 and 3.4 eV. In the following we argue that these peaks are due to antisymmetric and symmetric SP edge modes, which we label E_A_ and E_S_. These are indeed distinct peaks that do not evolve due to spectral shifts from the film mode peaks when moving across the film edge, as evidenced by a series of spectra shown in Supplementary Fig. 2 and discussed in Supplementary Note 1.

### Edge plasmon dispersion

**Figure 3:**
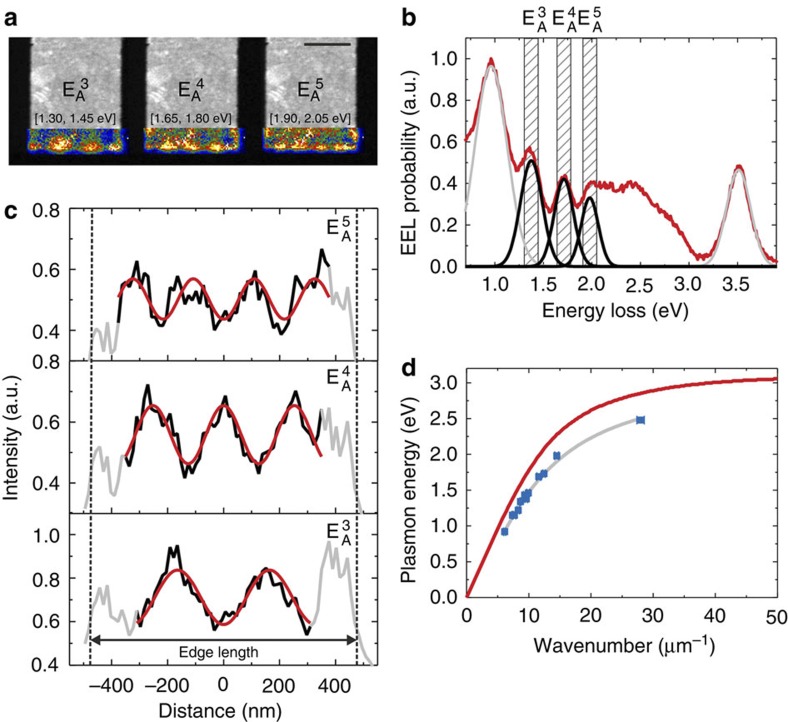
Plasmon modes of straight edges and their dispersion relation. (**a**) TEM image (scale bar, 500 nm) of a laterally structured 30-nm-thick silver film with a 0.95-μm-long lower edge superimposed with EEL maps acquired in the indicated energy ranges. The maps show standing wave patterns corresponding to antisymmetric plasmons of linear mode order *m*, denoted by 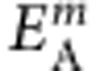. (**b**) EEL spectrum (red line) integrated along the lower film edge shown in **a**. The energy windows of the EEL maps in **a** are marked by the dashed boxes, the Gaussian curves (black lines) are fits to the experimental EEL peaks. (**c**) Intensity cross-cuts taken from **a**, the dashed lines mark the lateral film extension. The parts plotted in black (experimental data) are fit by a sinusoidal describing a standing wave (red lines). (**d**) Dispersion relation of edge plasmons (blue symbols), the grey line is a guide to the eye. The error bar size is discussed in detail in Supplementary Figs 3–5 and Supplementary Note 2. The red curve plots the calculated *F*_A_ dispersion relation for an extended 30-nm-thick silver film on a 15-nm-thick Si_3_N_4_ membrane.

As we now discuss for the E_A_ mode, the edge plasmon dispersion relation can be deduced by probing laterally confined film edges. The length of the edges was 0.3, 0.95, 1.25 and 1.55 μm while the film extension in the orthogonal direction was kept constant at 100 μm. Figure 3a exemplarily shows a TEM image of the 0.95-μm-long edge (for the other edge lengths see Supplementary Fig. 3) superimposed with EEL maps, revealing standing wave patterns due to edge plasmon reflection at the film corners. The energy ranges as indicated for each map were chosen from the spectrum in Fig. 3b that shows the EEL signal integrated along the edge. The individual peaks E_A_^*m*^ (*m* being the linear plasmon mode order) are fit with Gaussian functions and the energy windows for the EEL maps in Fig. 3a are indicated. Mode orders higher than 5 are not taken into account, because the corresponding peaks are energetically too close to be resolved experimentally. To extract the mode wavenumbers *k*_*m*_=2*π*/*λ*_*m*_ from the data, cross-cuts taken from the EEL maps are plotted in Fig. 3c and fit by sinusoidal functions describing standing waves with wavelength *λ*_*m*_. The vicinity of the lateral film boundaries (marked by the dashed lines) was excluded from the fitting procedure due to obvious edge- and reflection phase-induced effects distorting the signal. We now combine the data to a dispersion relation by plotting, with blue symbols in Fig. 3d, the peak energies retrieved from the fits in Fig. 3b versus the according wavenumber values (Fig. 3c). We note that the estimated measurement inaccuracies are smaller than the symbol size, as discussed in detail in Supplementary Figs 3–5 and Supplementary Note 2. The symbols trace a function that is significantly separated from the film dispersion relation (F_A_) plotted in red, which is calculated for an extended 30-nm-thick silver film on a 15-nm-thick Si_3_N_4_ membrane. The grey line is plotted as a guide to the eye only, as the implementation of the calculated edge mode dispersion^22^ goes beyond the scope of this paper. We interpret our results as evidencing a specific plasmon edge mode that in a confined geometry can be well described in a standing wave picture, along the lines of former work by Gu *et al*.^23^, Nelayah *et al*.^24^ and Novotny^25^.

### The case of nanodisks

**Figure 4:**
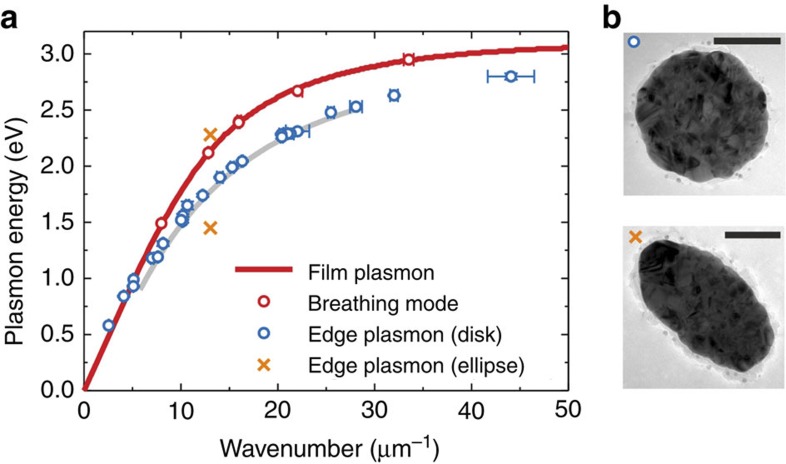
Nanodisk modes interpreted as edge and film plasmons. (**a**) Data points retrieved from the breathing modes and from the dipolar/multipolar modes of nanodisks are plotted by the red and blue symbols, respectively. The error bar size is discussed in detail in Supplementary Figs 3–5 and Supplementary Note 2. The nanodisks are 30 nm high with diameters of 100–800 nm, the substrate is a 15-nm-thick Si_3_N_4_ membrane. The red curve is the calculated dispersion relation of the *F*_A_ mode of a 30-nm-thick silver film on a 15-nm-thick Si_3_N_4_ substrate. The grey line is the guide to the eye copied from Fig. 3d. The orange symbols show the two in-plane dipolar excitations of an elliptical nanoparticle with axes lengths of 100 and 200 nm. (**b**) TEM bright-field images of both particle types are shown (scale bar, 100 nm).

Having identified the film and edge modes of a semi-infinite thin film, the question of the applicability of this concept to geometries of different dimensionality, in particular to nanoparticles smaller or comparable with the light wavelength, arises. Indeed, specific plasmonic excitations with radial symmetry (‘breathing’ modes) on silver nanodisks have recently been identified as antisymmetric film SPs confined to the disk geometry^18^. In this case the SP wavenumber is scaled by the radial confinement, with *k*_*n*_=2*π*/*λ*=2*πn*/*d*, where *λ* is the SP wavelength, *d* is the disk diameter and *n* is the radial mode order. Deducing the wavenumbers from disks with diameters in the 100–800 nm range (disk height 30 nm, substrate 15-nm-thick Si_3_N_4_) and combining them with the according EEL peak energies yield the data set plotted by the red symbols in Fig. 4. The data perfectly fit within measurement accuracy to the calculated dispersion relation of the antisymmetric SP (F_A_) of a 30-nm-thick film on a 15-nm-thick Si_3_N_4_ substrate^18^.

Besides the breathing modes, the nanodisks sustain dipolar and multipolar plasmon excitations. These modes are confined to the particle boundaries with a specific number of field antinodes along the particle circumference^18^. It is thus tempting to ask for their correspondence to the modes on the straight film edge we have analysed in Fig. 3. Accordingly, we assume the disk circumference *dπ* to determine the possible SP wavenumbers *k*_*ℓ*_=2*π*/*λ*=2*ℓ*/*d* for an angular mode order *ℓ*. The mode energies extracted from the peaks in the EEL spectra versus the so-defined wavenumbers are plotted in Fig. 4 as blue symbols. We observe that all data points closely follow the dispersion relation experimentally found for the E_A_ mode in Fig. 3c, from where the grey line in Fig. 4 is copied. We thus find that the dipolar and multipolar particle modes can be simply scaled to the plasmon modes of a straight edge. This result might appear unexpected at the first view. Generally, it could be expected that bent edges with a curvature radius as small as 50 nm would yield a dispersion relation different from that of a straight edge. In particular for small particles an influence of the geometry-dependent coupling of charges across the particle should play a role.

To shed light on this point, we apply a simple quasi 1D Drude-like model accounting for electrons confined to a straight or circular wire, as discussed in more detail in Supplementary Fig. 6 and Supplementary Note 3. For the straight wire and a given wavenumber *k*, the plasmon frequencies *ω* are given by the expression


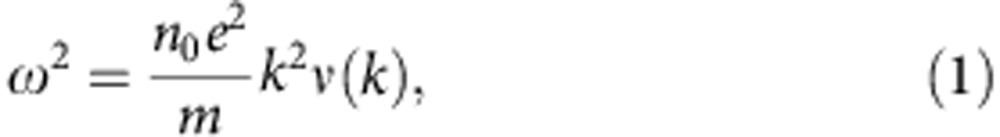


where *e* and *m* are the electron charge and mass, respectively, *n*_0_ is the electron density and *ν*(*k*)=2*K*_0_(*ak*) is the Fourier transform of the Coulomb potential, with *K*_0_ being the modified Bessel function of zeroth order. We have introduced a transversal extension *a* for the stripe-like wire to avoid divergences of *v*(*k*). For the circular wire with radius *R*, we can again use Equation (1) but have to replace *ν*(*k*) with


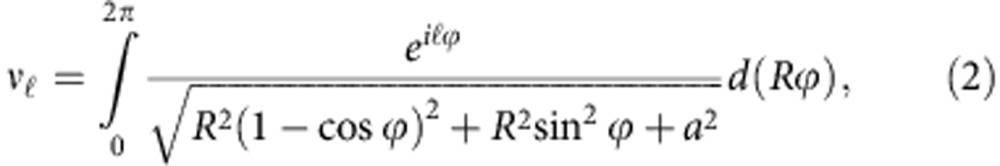


where we have introduced polar coordinates (*x*, *y*)=*R*(cos*ϕ*, sin*ϕ*) for the integration along the ring circumference. The enumerator accounts for the SP charge density wave with angular order *ℓ*, the denominator gives the distance from one point of the ring (here (*R*,0)) to the other points (*a* accounts again for effects of the transversal ring extension). Importantly, the dispersion relations for the straight and circular wire practically coincide, irrespective of the softness parameter *a* (for details see Supplementary Note 3). The ‘magic’ about the straight and circular wire geometries is that in both cases the charge distribution of the plasmonic modes is homogeneous, and the conserved quantities are linear and angular momentum, respectively. In transformation optics, one can map the problem of a thin metallic slab to a metallic cylinder^26^. Thus, although seemingly different, the two problems are intimately connected through a conformal mapping. The straight wire and ring SP modes of Equations (1) and (2) appear to be equally connected by some hidden symmetry. We note that things are markedly different for geometries with broken symmetry, as we illustrate with an elliptical nanoparticle with in-plane axes 100 and 200 nm long. In this case the two orthogonal dipole modes have different energies, and the corresponding data points (orange symbols in Fig. 4) do not comply with the edge plasmon dispersion relation.

### Symmetric modes

Besides the antisymmetric film and edge modes there exists a whole set of symmetric plasmonic modes for our sample geometries. In this case, however, detailed experimental evidence is lacking due to the increased damping at higher mode energies and the spectral overlaps with the bulk mode (Fig. 1). As the observed data indicates that the mode scaling turns out to be analogous to the antisymmetric case as expected, we study this point in a simulation analysis of the complete SP mode spectrum, as detailed in Supplementary Fig. 7 and Supplementary Note 4.

In summary, we have shown that plasmonic modes in thin-film-based (nano)structures can be scaled to film and edge modes. In particular, the mode dispersion of straight edges remains unchanged when large curvatures in the case of nanodisks are considered. This finding is intimately related to the homogeneous charge distribution in symmetric particles. Our results provide a simple ordering scheme of plasmon modes, fostering an intuitive understanding of the full mode spectrum in plasmonic systems and providing guidelines for their design.

## Methods

### Fabrication

Silver films 30 or 50 nm thick were thermally evaporated on 15-nm-thick Si_3_N_4_ membranes. Lateral structuring was done by electron-beam lithography in a RAITH e-line system using a standard lift-off process with a poly(methylmetacrylate) resist^18^. One sample was covered by an additional 22-nm-thick silicon dioxide layer deposited on top of the silver structures by electron beam evaporation.

### EELS measurements

EEL measurements were performed in a STEM (FEI Tecnai F20) with a monochromated 200 keV electron probe of 0.15 eV energy width (full-width-at-half-maximum). EEL spectra were measured with a high resolution Gatan Imaging Filter equipped with a charge-coupled device camera with 2,048 × 2,048 pixel and an energy dispersion of 0.01 eV per channel. The electron beam was scanned over the region of interest and spectra were acquired at each beam position (pixel)^27^. The scan step size in the spectrum images varied between 2.9 and 27 nm, the exposure times per EEL spectrum were chosen between 0.05 and 0.1 s. STEM images were acquired with a high-angle annular dark-field detector (Fischione Instruments). The EEL spectra were background subtracted by removing the tail of the zero-loss peak that includes directly transmitted and elastically scattered electrons by using a fitted logarithmic function (‘fitted logarithm tail’ model within Digital Micrograph software (Gatan, USA)).

### Simulations

Simulations were done with the MNPBEM toolbox^28^ that relies on the boundary element method approach^29^. The dispersion relations in Figs 1b, 3d and 4 were calculated with the transfer matrix method^30^.

## Author contributions

F.-P.S. fabricated the samples, performed the experiments and analysed the data. U.H., H.D. and A.H. performed the simulations and contributed to the data analysis. H.D., J.R.K. and F.H. designed the experiments and supervised the project. All authors contributed to writing the manuscript.

## Additional information

**How to cite this article:** Schmidt, F.-P. *et al*. Universal dispersion of surface plasmons in flat nanostructures. *Nat. Commun.* 5:3604 doi: 10.1038/ncomms4604 (2014).

## Supplementary Material

Supplementary Information Supplementary Figures 1-7, Supplementary Notes 1-4 and Supplementary References
