# Supplementary material for: Universal dispersion of surface plasmons in flat nanostructures
Source: Nat Commun. 2014 Apr 10;5:3604. doi: 10.1038/ncomms4604 (PMC4071950; doi:10.1038/ncomms4604)
Supplement: Supplementary Information — Supplementary Figures 1-7, Supplementary Notes 1-4 and Supplementary References [file ncomms4604-s1.pdf]

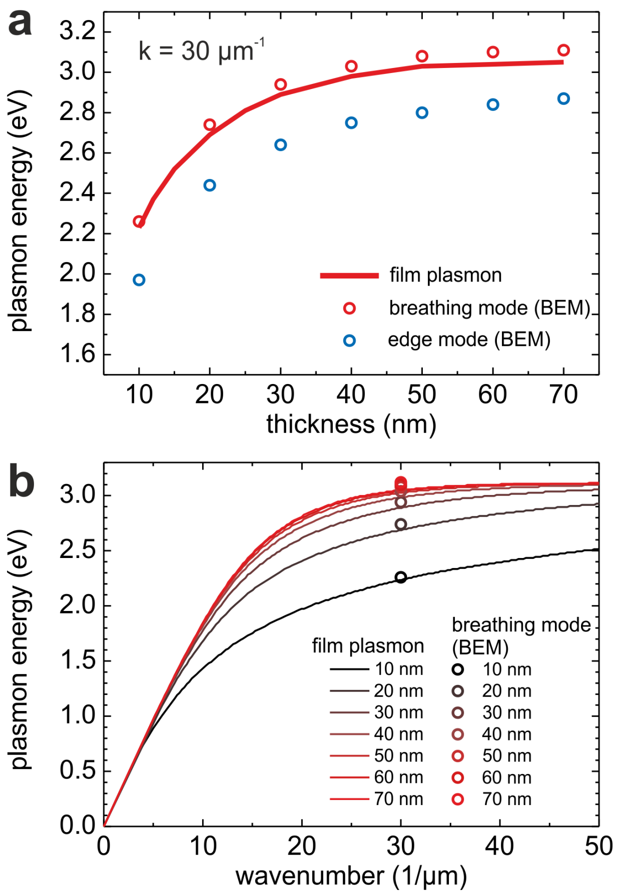

Supplementary Figure 1 | Simulations of the film thickness dependence of plasmon modes on films or disks on a 30 nm thick Si_3N_4 substrate. (**a**) Simulated plasmon energy at k=30 μm^{-1} for the surface plasmon (line), the disk breathing mode (red symbols) and the disk edge mode (blue symbols). (**b**) Plasmon dispersions for 10-70 nm thickness as defined in the legend for the surface plasmon (lines) and the disk breathing mode (symbols). The silver film and disk thickness range of $\geq$ 30 nm as considered in the main text gives rise to only weak coupling between adjacent interfaces or edges. While the plasmon energies strongly decrease for film thicknesses smaller than 30 nm, the difference of, e.g., the film mode energy between this thickness value and a single interface (infinitely thick film) is only 0.21 eV. The according difference between thicknesses of 30 and 50 nm is 0.16 eV. It is important to note that these energy differences are not the values observed in the measurements (Figs. 1 and 2 in the main text), as the EELS peaks correspond to the asymptotic branches of the dispersion relations. Here, the differences are much smaller as evident from the the dispersion relations plotted in **b**.

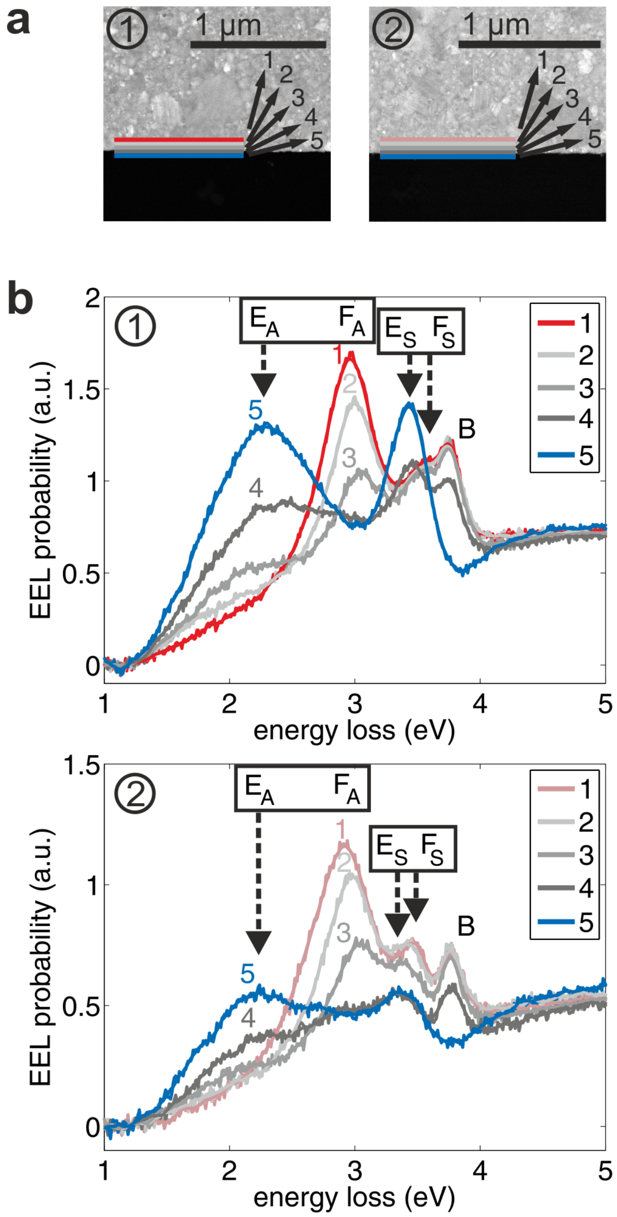

Supplementary Figure 2 | Film vs. edge plasmons (extended version). (**a**) TEM images of 30 nm thick silver films with lithographically defined edges, (1) without and (2) with a 22 nm thick SiO_2 cover layer. The spectra in **b** have been integrated along the full lines in the TEM images. (**b**) EEL spectra acquired along the same-colored lines in **a** for samples (1) (top) and (2) (bottom). The spectra were acquired within the film up to distances to the edge of 45 nm [red spectrum in (1)] and 60 nm [pink spectrum in (2)], on the edge and on the substrate side at a distance of 15 [blue spectrum in (1)] and 20 nm [blue spectrum in (2)].

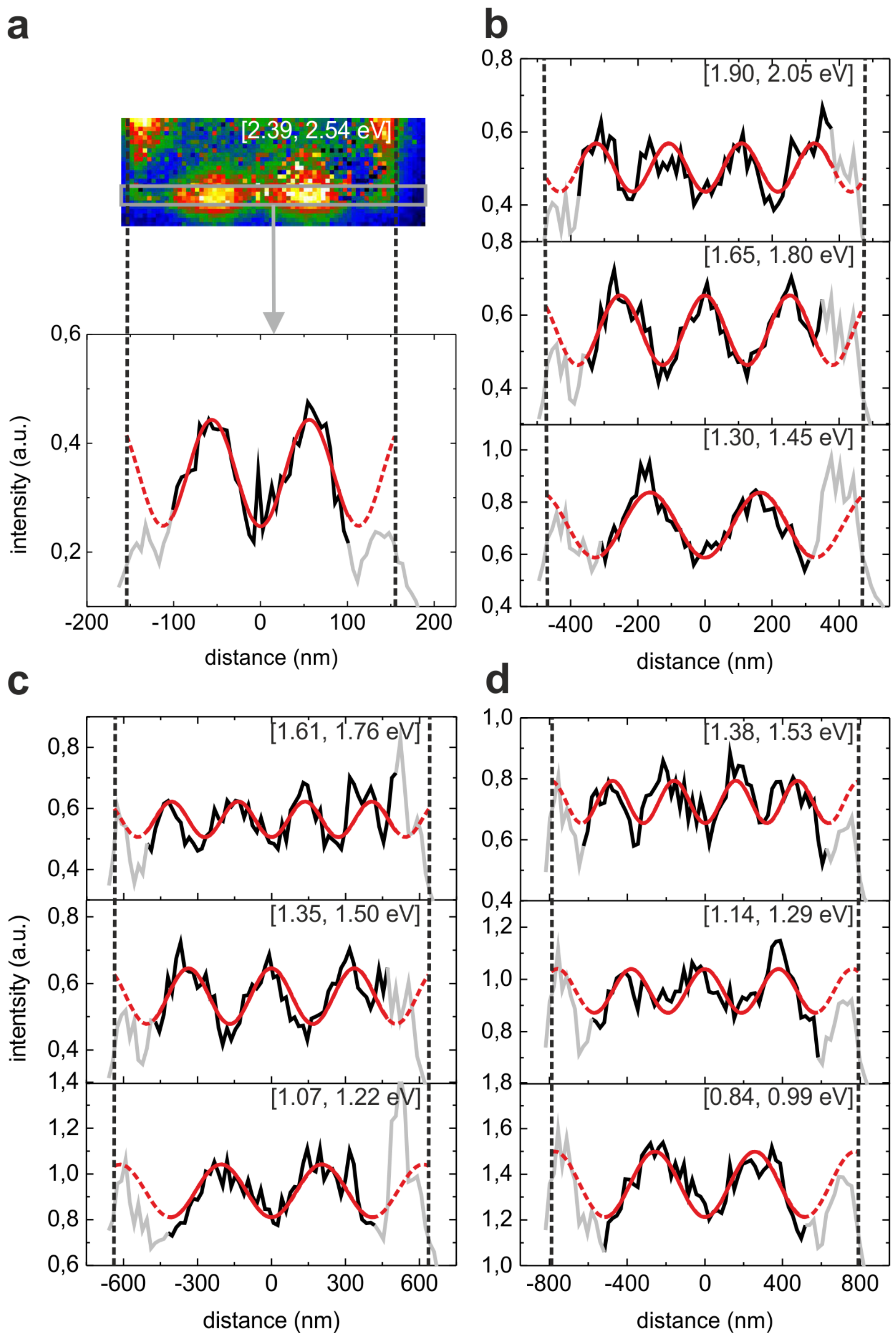

Supplementary Figure 3 | Retrieving SP mode wavenumbers on straight film edges. (**a**) The EEL map (on top) was acquired around a 300 nm long straight silver edge in the indicated energy range. The cross-cut plotted below is averaged over the area between the two horizontal grey lines in the EEL map. The parts plotted in black (experimental data) are fit by a sinusoidal describing a standing wave (red line). (**b**)-(**d**) EEL map cross-cuts for edge lengths of 0.95, 1.25 and 1.55 μm. The dashed lines mark the lateral film edges.

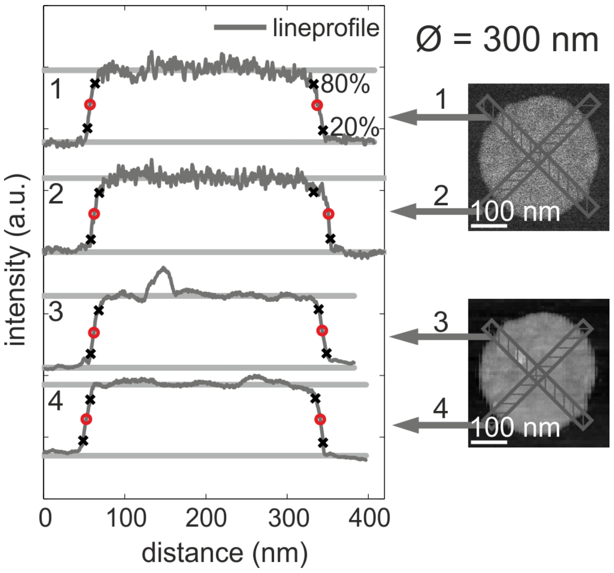

Supplementary Figure 4 | Measurement of nanodisk diameter. Line profiles extracted from STEM images taken before (1,2) and during spectrum image acquisition (3,4) of a silver nanodisk. The disk diameters at half disk-induced signal intensity (red symbols) and the distances between the 20% and 80% values (black crosses) of the signal maximum were considered to retrieve the mean disk diameter and mean error, respectively.

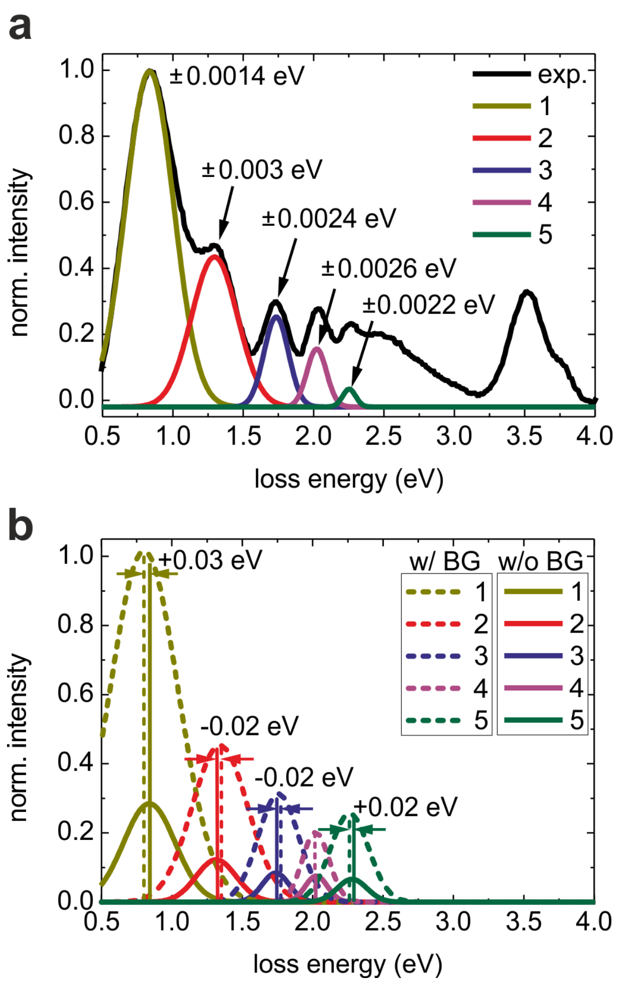

Supplementary Figure 5 | Determination of peak positions. (**a**) The experimental spectrum (black curve) is fitted after zero loss subtraction by Gaussian functions (1-5). The standard errors of peak positions retrieved from the fitting procedure are displayed. (**b**) Difference of fitted peak positions before (dashed spectra) and after zero loss subtraction (solid lines) is shown.

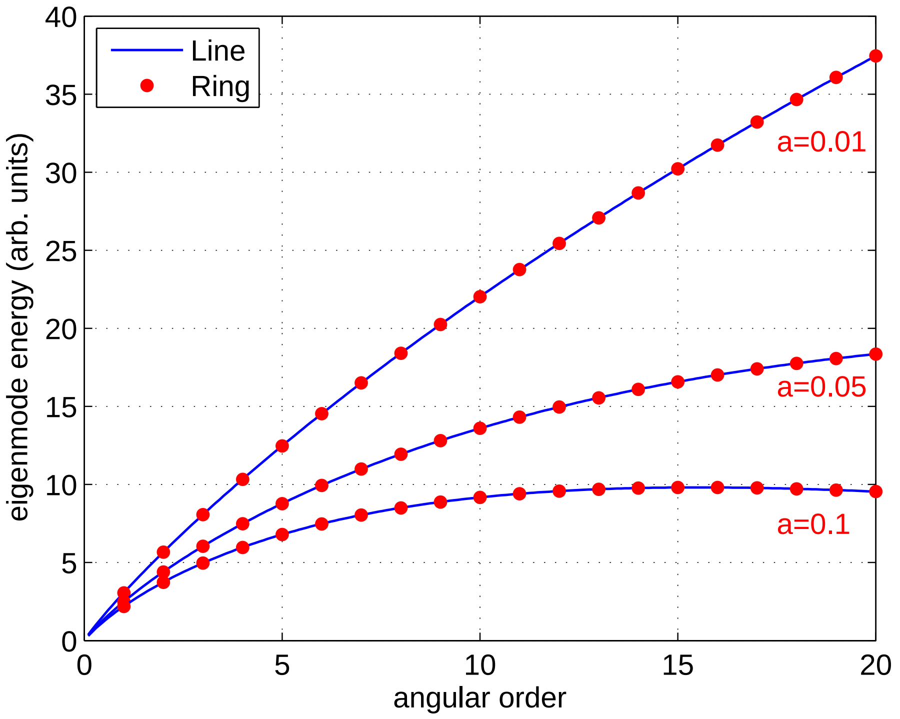

Supplementary Figure 6 | Drude-type model. Plasmonic eigenmodes for quasi one-dimensional straight (solid line) and circular (red symbols, $R = 1$) wire, as computed from Drude-type model, and for different softness parameters a. For details see Supplementary Note 3.

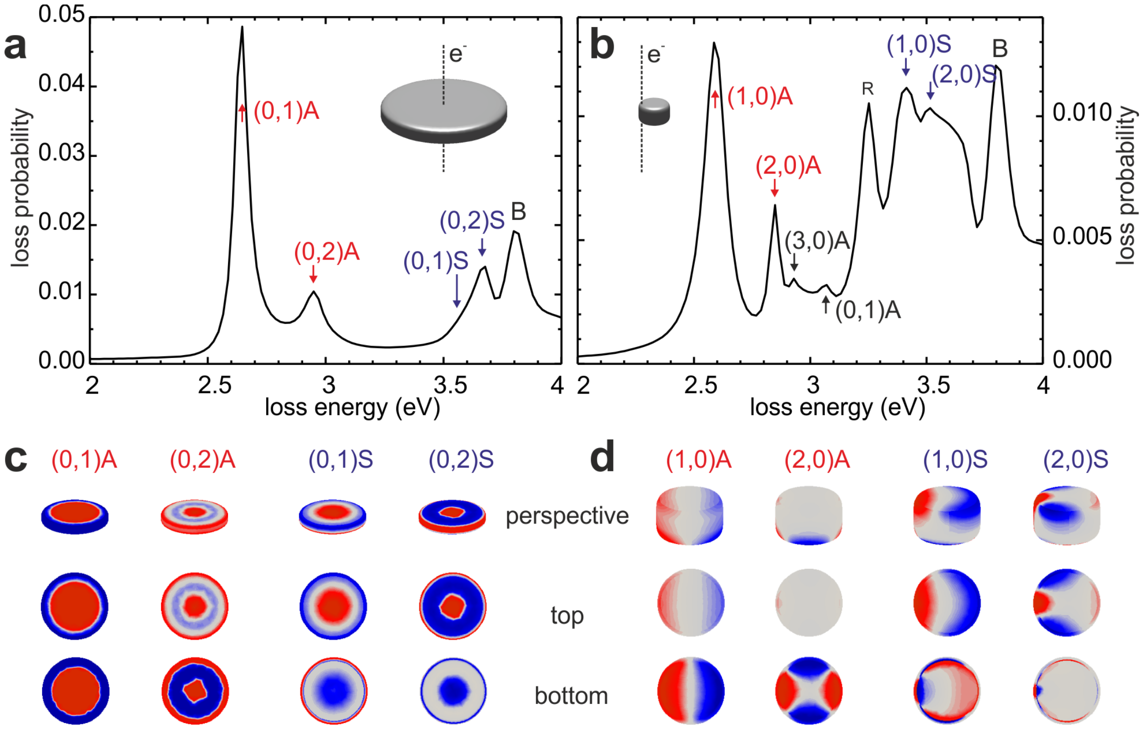

Supplementary Figure 7 | BEM simulation on silver nanodisks. (**a**) EEL spectra calculated of a 30 nm thick silver nanodisk, 200 nm in diameter, on a 30 nm thick Si_3N_4 substrate for an electron beam position in the center of the disk (see inset). (**b**) The disk diameter is reduced to 50 nm and the electron beam position is set 3 nm apart from the disks' edge on the disk (see inset), while the other parameters are the same as in **a**. The spectrum shows peaks corresponding to antisymmetric (red) and symmetric (blue) edge modes. (**c**) Surface charge distributions for plasmonic eigenmodes corresponding to the four peaks $(0,1)$A, $(0,2)$A, $(0,1)$S and $(0,2)$S shown in **a**. Three different angle of views are presented (perspective, top and bottom view), while the red color corresponds to positive charge and blue color to negative charge respectively. (**d**) Surface charge distributions for edge modes $(1,0)$A, $(2,0)$A, $(1,0)$S and $(2,0)$S corresponding to the equally labelled peaks in **b**.

Supplementary Note 1: Film and edge plasmons

Complementing the spectral data in Fig. 2b we summarize in Supplementary Figure 2 further EEL spectra taken at different distances from the film edge. Each spectrum in Supplementary Figure 2b is integrated along the the same-color line depicted in the TEM images in Supplementary Figure 2a. The red and blue curves in Supplementary Figure 2b (upper panel) correspond to the spectra plotted in Fig. 2, acquired on the film side 45 nm away from the film edge and on the substrate side at a distance of 15 nm, respectively. The grey curves depict the spectra taken for positions in between, with a distance increment of 15 nm. We observe that when moving over the edge to the bare substrate a steady decrease of the film plasmon peaks F_A (3.0 eV) and F_S (3.6 eV) is observed that is accompanied by an increase of the edge peaks E_A (2.3 eV) and E_S (3.4 eV). The bulk plasmon peak B vanishes when the electron beam moves off the film. This series of spectra illustrates that the film and edge peaks are due to distinct excitations and do not evolve from each other due to spectral shifts.

In addition, we did the same set of measurements for the 30 nm thick silver film covered with a 22 nm thick SiO_2 layer (compare Fig. 1). The according spectra are summarized in the lower panel of Supplementary Figure 2b. In this case the symmetric film and edge plasmon peaks are shifted to lower energies and are thus more clearly separated from the bulk peak B. Again, the pairs of film and edge modes appear as distinct peaks.

Supplementary Note 2: Measurement accuracy

Our findings discussed in the main text rely on EEL data with rather small error margins that enable us to retrieve distinct dispersion relations for film and edge plasmon modes. Here, we address this point by discussing the limiting factors for, first, experimentally determining the SP wavenumber and, second, for measuring the EEL peak energy that together set the error bars in Fig. 3 and 4 of the main text.

The SP modes on the laterally confined straight edges form standing waves of order m. Cross-cuts through the EEL maps allow the direct retrieval of the SP wavelength λ_m and thus the wavenumber $k_m = 2\pi/\lambda_m$, as shown in Fig. 3 of the main text for an edge length of $0.95\,\mu$m. In Supplementary Figure 3a-d we additionally include the data for edge lengths of $0.3, 1.25$ and $1.55\,\mu$m that were all used to derive the dispersion relation in Fig. 3d. The standing waves were fit by sinusoidal functions, yielding λ_m and the corresponding standard error that was calculated using the Levenberg-Marquardt algorithm in a nonlinear regression (Origin, OriginLab, Northampton, MA). Reliable fitting was not achieved on modes of first and second order which were thus not considered. The data range close to the film corners (plotted in grey) was excluded from the analysis due to distorting edge effects.

Nanodisk SP modes are either "breathing" excitations, described by $k_n = 2\pi/\lambda_n = 2\pi n/d$ (d disk diameter, n radial mode order) or of dipolar/multipolar character with $k_\ell = 2\pi/\lambda_\ell = 2\ell/d$ (ℓ angular mode order). The wavenumber error is thus given by the accuracy of measuring the disk diameter. For each disk (100, 200, 300, 400, 500 and 800 nm in diameter) line profiles along different radii were extracted from STEM images and a mean diameter with a standard deviation was derived. In Supplementary Figure 4 this is shown for the disk of 300 nm diameter. Line profiles along two perpendicular directions were taken from the marked regions, considering two images acquired before and during the spectrum image acquisition to improve statistics. The diameter was taken at the half-height of the maximum disk-induced signal intensity in the cross-cuts, marked by the red symbols in Supplementary Figure 4. The error was defined as the lateral distance between between the 20% and 80% signal intensity values (black crosses in Supplementary Figure 4). The mean values of all four measured diameters and corresponding errors were used to determine wavenumber and wavenumber error, respectively. In summary, the evaluated wavenumber errors are ranging between 0.02 μm^{-1} for the largest and 2.4 μm^{-1} for the smallest disk, that correlates to 0.6 - 5.5% deviation of the mean value.

We now turn to the limiting factors for energy resolution, i.e., for measuring peak positions. The energy spread of the exciting electron beam was symmetric with a full-width-at-half-maximum of 0.15 eV. The channel width of the spectrometer was 0.01 eV. Four contributions can potentially decrease the peak position accuracy. First, we use Gaussian fits to find the EEL peak positions in the experimental spectra, as exemplarily illustrated in Supplementary Figure 5a for a disk with a diameter of 500 nm. The fit errors that are again calculated with the Levenberg-Marquardt algorithm in a nonlinear regression (Origin, OriginLab, Northampton, MA) are in any case clearly below the spectrometer channel width of 0.01 eV and thus introduce no further resolution limitation. Second, we consider an error contribution from the zero-loss peak subtraction which is expected to be more pronounced at low energies, especially below 0.5 eV. To estimate the resulting error we fit Gaussian peaks to the spectra (again from 500 nm diameter disk) without (dashed curves in Supplementary Figure 5b) and with zero-loss subtraction (solid curves in Supplementary Figure 5b). As expected the difference and therefore the impact of the zero loss subtraction is higher for lower peak energies. For example, for the case illustrated in Supplementary Figure 5b the energy amounts to about 0.03 eV. Third, the imperfect shape of lithographed structures can limit our energy accuracy as peak energies of surface and edge plasmons are very sensitive to size and shape of the nanostructure. In particular, deviations from the nominally circular cross-section of nanodisks might lead to a lifting of the plasmon mode degeneracy and thus to inhomogeneous spectral broadening. We estimate this standard error contribution to around 0.04 eV by measuring peak energies on different positions along the disk edge. Fourth, the calibration of the spectrometer itself can limit the energy precision. For the calibration of the used dispersion

(0.01 eV/channel) the rather sharp aluminum plasmon peak was used and the according error is estimated to below 0.025 eV. Additionally the bulk plasmon peak of silver at 3.75 eV was used as a calibration marker in each experiment. In summary, we estimate the energy error to be smaller than 0.05 eV, a value clearly below the symbol size in Fig. 3 and 4 in the main text.

Supplementary Note 3: Drude-type model

For the hydrodynamic plasmon model we follow Mendoza et al. [3] and assume, for a given external potential, a semiclassical equation of motion for the induced charge distribution $\delta\rho$,

$$\frac{d^2\,\delta\rho(\boldsymbol{r},t)}{dt^2}=\frac{n_0e^2}{m}\,\nabla^2\phi(\boldsymbol{r},t)\,, \tag{1}$$

where e and m are the electron charge and mass, respectively, and n_0 is the electron density. The Coulomb potential

$$\phi(\boldsymbol{r},t)=\phi_{\rm ext}(\boldsymbol{r},t)+\int\frac{\delta\rho(\boldsymbol{r}',t)}{|\boldsymbol{r}-\boldsymbol{r}'|}\,d\tau' \tag{2}$$

consists of an external and induced part, respectively. Eigenmodes correspond to solutions in absence of an external excitation, which leads after Fourier transform to the mode equation [see also Eq. (1) of main manuscript]

$$\omega^2=\frac{n_0e^2}{m}k^2\nu(k)\,. \tag{3}$$

$\nu(k)$ is the Fourier transform of the Coulomb potential. For a one-dimensional wire and a soft Coulomb potential, accounting for wire extensions in the transversal directions, we obtain

$$\nu(k)=\int_{-\infty}^{\infty}\frac{e^{ikx}}{\sqrt{x^2+a^2}}\,dx=\int_{-\infty}^{\infty}\frac{\cos kx}{\sqrt{x^2+a^2}}\,dx=2\,K_0(ka)\,. \tag{4}$$

Note that a is needed to avoid divergencies of the Coulomb potential. A completely similar approach can be pursued for the ring-like wire, but the one-dimensional Fourier transform for $\delta\rho$ has to be replaced by an expansion in terms of angular orders $\delta\rho(\varphi)=\sum_\ell e^{i\ell\varphi}\delta\rho_\ell$. Correspondingly, $\nu(k)$ has to be replaced by the expression given in Eq. (2) of the main manuscript. In Supplementary Figure 6 we plot the dispersion relations for the straight and circular wire geometries, and for different softness parameters a. We relate the order ℓ of the angular modes to the wavenumber ℓ/R, where R is the ring radius. As can be seen, the two dispersion curves lie almost on top of each other.

Supplementary Note 4: Simulation of symmetric film and edge modes

In the main text we have illustrated the plasmon mode scaling with the antisymmetric film and edge modes. While we come to the same findings by analyzing the symmetric modes this analysis suffers from significantly weaker signals due to stronger damping at higher loss energies and spectral overlaps, in particular with the bulk plasmon. To unravel the full picture we thus turn to a simulation analysis of the complete mode spectrum based on the boundary element method (BEM) [1] and using a dielectric function of silver extracted from optical experiments [2]. For the film modes we calculated the EEL spectrum of a silver disk (200 nm in diameter and 30 nm thick) placed on a Si_3N_4 substrate (modelled as an additional disk with 345 nm in diameter and 30 nm thick). The electron beam transverses the disk center, as sketched in the inset of Supplementary Figure 7a. The according EEL spectrum (Supplementary Figure 7a) shows four peaks, one of them having a weak shoulder. In the following we classify the modes by (n, ℓ) describing their radial mode order n and angular mode order ℓ, followed by A or S to indicate the antisymmetric or symmetric mode character. By plotting simulated maps of the surface charges at the peak (and shoulder) energies in Supplementary Figure 7c, we see that the dominant peak at 2.65 eV corresponds to the first order antisymmetric breathing mode $(0, 1)$A with a symmetric surface charge distribution at the top and bottom interfaces. The same distribution pattern is found for the second order antisymmetric breathing mode $(0, 2)$A that as well shows a distinct peak in the EEL spectrum. In contrast, the EEL peak at 3.68 eV and its shoulder at 3.55 eV are found to be due to the symmetric plasmon modes $(0, 2)$S and $(0, 1)$S, respectively, with antisymmetric charge distributions at the top and bottom interfaces of the nanodisk. Both of these resonances are weak, probably because their energies are close to the asymptotic energy of the symmetric SP, resulting in strong damping and strongly localized fields with weak coupling to the electron. We note that the peak 3.68 eV does not shift noteworthy when changing the disk diameter. Thus, we interpret it as the asymptotic peak of the symmetric surface plasmons (corresponding to the experimental peak F_S in Fig. 2b) weakly enhanced by the $(0, 2)$A resonance. Again, B is the bulk plasmon peak.

Things are quite similar for the edge modes. We show this at a disk with reduced diameter (50 nm instead of 200 nm) because the symmetric edge mode is even higher damped than the symmetric film mode. In the EEL spectrum, calculated for an electron beam penetrating the disk 3 nm apart from its edge (Supplementary Figure 7b), the first three peaks (starting on the low energy side) correspond to the dipole $(1, 0)$A, quadrupole $(2, 0)$A, and hexapole $(3, 0)$A antisymmetric edge modes. This interpretation is corroborated by the symmetry of the surface charge maps of dipole and quadrupole presented in Supplementary Figure 7d. The dipole $(1, 0)$S and hexapole $(2, 0)$S peaks

caused by the symmetric edge mode appear at 3.4 eV and 3.5 eV. At least for the dipole, the antisymmetry of the surface charges on top and bottom of the disk is clearly visible in Supplementary Figure 7d. The distortion of the dipole as well as the quadrupole pattern is due to the high damping of the mode and the position of the electron beam close to the disk edge. For the sake of completeness, we attribute the peak labelled $(0, 1)$A to the first order antisymmetric breathing mode, the peak R to the vertical dipole mode (first order rod mode), and again the peak B to the volume plasmon.

Supplementary References

[1] Hohenester, U. & Trügler, A. MNPBEM - A Matlab toolbox for the simulation of plasmonic nanoparticles. *Comp. Phys. Commun.* **183**, 370–381 (2012).

[2] Johnson, P. B. & Christy, R. W. Optical constants of the noble metals. *Phys. Rev. B* **6**, 4370–4379 (1972).

[3] Mendoza Santoyo, B. & del Castillo-Mussot, M. Plasmons in three, two and one dimension. *Rev. Mex. Fis.* **39**, 640–652 (1993).
